# Absorption of Nutriment Injected Subcutaneously

**Published:** 1869-08

**Authors:** 


### Absorption of Nutriment Injected Subcutaneously.

Drs. Menzel and Perco, in Vienna, seem entitled to the credit of some originality, and perhaps of a useful discovery; though it were premature to speak of the practical value of their researches. They began with the injection of almond, olive and cod-liver oils under the cutis of dogs—the quantity used varying from a drachm to an ounce. Finally, after twenty-five successful experiments of this kind, they obtained Prof. Billroth's consent to practice a similar injection upon one of his patients, the quantity injected in the latter case being 9 gr. (grammes?); and with the same successful result as that obtained in the other case, namely, absorption of the oil in from 36 to 48 hours, without any local inflammation, or any other evil consequences. Returning to their *corpora vilia* they now injected a drachm of milk once; one or two drachms of syrup simplex ten times, and a drachm of the yolk of a hen's egg four times, with perfect success in each case, the substances being usually absorbed complete within twenty-four hours.

Strickner and Oser have practised similar injections of peptone upon patients—but the trial of undigested nutriments has not, it is claimed, been previously made.—*Wiener Med. Wochensch, and Boston Med. & Sur. Jour.*

